# Calcium Channels and Calcium-Binding Proteins

**DOI:** 10.3390/ijms241814257

**Published:** 2023-09-19

**Authors:** Sumiko Mochida

**Affiliations:** Department of Physiology, Tokyo Medical University, Tokyo 160-8402, Japan; mochida@tokyo-med.ac.jp

Signals of nerve impulses are transmitted to excitatory cells to induce the action of organs via the activation of Ca^2+^ entry through voltage-gated Ca^2+^ channels (VGCC), which are classified based on their activation threshold into high- and low-voltage activated channels, expressed specifically for each organ. Ca^2+^ entering into excitatory cells binds to Ca^2+^-binding proteins, which are classified into two groups of Ca^2+^ sensors: C2 domain and EF hand proteins. Within transient Ca^2+^ elevation, each Ca^2+^ sensor, having different affinity and binding speeds to Ca^2+^, determines the timing of protein–protein interactions to produce the specific action of each organ. This Special Issue aims to focus on investigations ranging from the control of Ca^2+^ entry at the plasma membrane of excitatory cells to detailed studies on the intracellular mediation of Ca^2+^-binding proteins that catalyze organ-specific action. The submitted papers are studies of Ca^2+^ channels and Ca^2+^ dynamics controlled by Ca^2+^ elevation in neurons, cardiomyocytes and osteosarcoma. Thus, recent findings of Ca^2+^ channels’ regulation and Ca^2+^ dynamics controlled by Ca^2+^ elevation in neurons are discussed before summarizing the papers in this Special Issue.

In the nervous system, VGCCs are locally activated in different populations of neurons and subcellular compartments [1]. A certain fraction of the total VGCC population expressed in the membrane is composed into signaling complexes, with their signaling capacity being dependent on kinetic properties and their interaction with intracellular binding partners. However, another recently proposed possibility for the regulation of the distribution and efficiency of Ca^2+^ transients is a change in the local density of VGCCs [1]. For example, the activity-dependent regulation of the postsynaptic spine apparatus of hippocampal neurons depends on the activation of the perisynaptic L-type VGCCs and ryanodine receptors (RyRs) in the endoplasmic reticulum (ER) membrane, to induce Ca^2+^ induced Ca^2+^ release (CICR), and Ca^2+^-sensing stromal interaction molecule 1 (STIM1), a component of ER, which leads to the inactivation of L-type VGCC within seconds to minutes, resulting in more effective Ca^2+^ entry from the N-methyl-d-aspartate receptors (NMDARs) in spines (Figure 1A) [2]. In the suprachiasmatic nucleus (SCN), neuron changes in the density of dendritic L-type VGCCs during the circadian rhythm induce an alternation in CICR, which has consequences for the activation of large conductance Ca^2+^-activated K^+^ channels (BK channels) and controls the neuronal excitability (Figure 1B) [3]. Fast interactions in the order of a few milliseconds are effective for the communication between Ca^2+^ nanodomains of somatic P/Q-type VGCCs activating CICR and trigger the opening of BK channels, which affects the excitability of the somatic compartment of cartwheel interneurons (Figure 1C) [4]. BK or KCa1.1 channels are members of the Ca^2+^-activated potassium (KCa) channel family [5]. Within the KCa channel family, BK channels are the only member activated by both voltage and an increase in intracellular Ca^2+^ concentration [Ca^2+^]_i_ ≥ 10 µm [6]. In presynaptic terminals, pore-forming α1 subunits of Ca_V_ channels are also associated with BK channels via RIM-binding proteins/RIM [7], major active zone proteins. The BK channel N terminus is an endogenous ligand of the auxiliary Ca_V_α_2_δ subunit, displacing Ca_V_α_2_δ from the Ca_V_ channel complex at the plasma membrane [8]. Neurotransmitter release is precisely controlled by the time window of Ca^2+^ elevation in the synaptic vesicle release site.

Membrane–membrane contacts controlling Ca^2+^ signaling are a complex built between L-type VGCCs at the plasma membrane and RyRs in the ER, well known from muscle cells [9,10]. Store-operated Ca^2+^ channels (SOCCs), also known as calcium release-activated Ca^2+^ channels [11], consist of calcium-sensing stromal interaction molecules (STIMs) within the ER membrane and pore-forming Orai proteins in the plasma membrane [12]. STIM1 is a transmembrane protein originally described in immune cells [13] that plays a key role as a main activator of store-operated channels and membrane-associated calcium sensors in the ER [14]. STIM1 also binds directly to the C-terminus of the Ca_V_1.2 α1 subunit, suppressing their depolarization-triggered opening (Figure 1A) and inducing their internalization [15,16]. Recently, adaptor proteins such as SH3 and cysteine-rich containing proteins (STAC proteins) have been found as molecules that form a molecular complex between VGCCs in the plasma membrane and RyRs in the ER membrane, and participate in the coordination of CICR [17,18].

Ca^2+^ transients are terminated calcium pumps which ensure low [Ca^2+^]_i_ in the cytosol and are efficient in controlling sub-membrane calcium concentrations [19]. In excitable cells, the active transport of Ca^2+^ against the ion gradient is carried out primarily by the Na^+^/Ca^2+^ exchanger (NCX) and the plasma membrane Ca^2+^-ATPase (PMCA). A dynamic association of PMCAs and VGCCs mediates the fine-tuning of local Ca^2+^ domains and the regulation of [Ca^2+^]_i_ via Ca^2+^-mediated communication between STIM proteins in the ER membrane and VGCCs in the plasma membrane [1].

The piriform cortex (PC) is a central component of olfactory information processing, underlying odor discrimination and contextualization [20]. The PC, situated in the ventrolateral forebrain, has a laminar structure consisting of layers of pyramidal and semilunar neurons (layers II and III) whose dendrites extend out to the lateral olfactory tract (LOT) near the tissue surface, forming synaptic connections in adjacent layers. The inner synaptic layer (Ib) consists of associational cortico-cortical synapses, while the outer layer (Ia) makes synaptic connections with the LOT, passing afferent olfactory information via fibers from the olfactory bulb [21]. In conjunction with its role in olfactory encoding, the PC is a site involved in associative memory formation, exhibiting a high degree of synaptic plasticity during early developmental periods, which moderately persists into adulthood [22,23,24]. Similar to the hippocampus, the high- and low-frequency stimulation of afferent and associational fibers within the PC produce patterned Ca^2+^ influx via NMDARs to induce the long-term potentiation (LTP) or long-term depression (LTD) of synaptic activity, respectively [22,23,24]. NMDARs and voltage-gated L-type calcium channels (LTCCs) initiate diverse Ca^2+^-dependent signaling cascades that underlie the molecular basis of associative memory across different developmental stages. In this Special Issue, Rajani et al. demonstrated age-dependent changes in synaptic plasticity [25]. They investigated the expression and contribution of NMDARs and LTCCs in LTD of the PC associational fiber pathway in three cohorts of Sprague Dawley rats: neonatal (1–2 weeks), young adult (2–3 months) and aged (20–25 months). Using a combination of slice electrophysiology, Western blotting, fluorescent immunohistochemistry and confocal imaging, a shift from an NMDAR to LTCC mediation in LTD in aged rats was observed, despite there being no difference in the amount of LTD expression. These changes in plasticity are related to age-dependent differential receptor expression in the PC. LTCC Cav1.2 expression relative to postsynaptic density protein 95 was increased in the associational pathway of the aged PC layer Ib. Enhanced LTCC contribution in synaptic depression in the PC may contribute to altered olfactory function and learning with aging. These results suggest age-dependent contributions of NMDAR and LTCC to LTD in the PC.

The suprachiasmatic nucleus (SCN) is the central clock that coordinates peripheral oscillators to control circadian rhythms in mammals [26]. Photic cues, conveying information from the retina to the SCN via the glutamatergic retinohypothalamic tract, produce biphasic phase shifts, with delays in the early night and advances in the late night, during the dark phase of the light-dark cycle [27]. The glutamate-induced phase shifts involve Ca^2+^ entry, intracellular Ca^2+^ signaling mechanisms, gene expression, and protein synthesis [28,29]. In this Special Issue, Cheng et al. [30], using a ratiometric Ca^2+^ and Na^+^ imaging technique, investigated glutamate-evoked intracellular Ca^2+^ signaling that mediates the photic entrainment of the central clock in the SCN. The application of glutamate (100 µM) or high (20 mM) K^+^ induced an increase in [Ca^2+^]_i_. The Ca^2+^ clearance of the glutamate-induced Ca^2+^ transient was slower than that of the high K^+^-induced Ca^2+^ transient, and followed by the Ca^2+^ rebound. The time course of the Ca^2+^ clearance and the Ca^2+^ rebound depended on the duration of glutamate exposure. The application of glutamate, but not high K^+^, increased [Na^+^]_i_. In addition, the Ca^2+^ rebound was abolished by ouabain, which inhibits Na^+^/K^+^-ATPase (NKA), monensin, which increases Na^+^ by activating NKA, Na^+^-free solution, or nimodipine, which blocks L-type channels. These results suggest that glutamate-induced Ca^2+^ rebound originates from Na^+^ loads through activated NKA. Ouabain or Na^+^-free solution also slow Ca^2+^ clearance, apparently by retarding the Na^+^/Ca^2+^-exchanger (NCX)-mediated Ca^2+^ extrusion. Thus, the time cause of the glutamate-evoked Ca^2+^ response is controlled by glutamate-induced Na^+^ loads and NKA and NCX activation. The glutamate-activated NKA promotes Na^+^ extrusion and mediates rebound Ca^2+^ suppression, which accelerates Ca^2+^ clearance. In the absence of external Na^+^, Ca^2+^ clearance is still slower for the Ca^2+^ response to glutamate than for high K^+^, suggesting the participation of additional Ca^2+^ handlers in the slower Ca^2+^ clearance under this condition.

The soma, dendrites, and axon of neurons may display the Ca^2+^-dependent release of transmitters and peptides. Such a release is named extrasynaptic because it occurs in the absence of synaptic structures. In this Special Issue, De-Miguel reviewed the cooperative actions of three Ca^2+^ sources on somatic exocytosis [31]. The somatic release of serotonin was investigated using the classical leech Retzius neuron, which allowed detailed studies on the fine steps from excitation to exocytosis. Trains of action potentials induced transmembrane Ca^2+^ entry through L-type VGCCs. For action potential frequencies above 5 Hz, the summation of Ca^2+^ transients on individual action potentials activated the second calcium source: RyRs produced CICR. The resulting Ca^2+^ tsunami activated mitochondrial ATP synthesis to fuel the transport of vesicles to the plasma membrane. Serotonin released from the vesicles maintained a large-scale exocytosis by activating the third Ca^2+^ source; serotonin autoreceptors coupled to phospholipase C promoted inositol 1,4,5-trisphosphate (IP3) production. Activated IP3 receptors in peripheral ER released Ca^2+^ that promotes vesicle fusion. The machinery for somatic exocytosis has a striking disadvantage. The essential Ca^2+^ releasing ER near the plasma membrane prevents vesicle transport, drastically reducing the thermodynamic efficiency of the ATP expenses and elevating the energy cost of release.

Ca^2+^ is a critical second messenger in almost all cell types [32]. Likewise, Ca^2+^ plays a central role in the nervous system, where it is closely linked to the regulation of numerous neuronal functions such as neurotransmission, neuronal excitability, or gene expression [33]. On the other hand, an excessive increase in Ca^2+^ levels can also activate a series of harmful mechanisms for the cell, such as alterations in mitochondrial functioning and the generation of free radicals, which can eventually cause cell death through apoptosis [34]. Pesticides of different chemical classes exert their toxic effects on the nervous system by acting on the different regulatory mechanisms of Ca^2+^ homeostasis. Pesticides have been shown to alter Ca^2+^ homeostasis, mainly by increasing its intracellular concentration above physiological levels. Pesticide-induced Ca^2+^ overload occurs through two main mechanisms: the entry of Ca^2+^ from the extracellular medium through the different types of Ca^2+^ channels present in the plasma membrane, or its release into the cytoplasm from intracellular stocks, mainly from the ER. It has also been observed that intracellular increases in Ca^2+^ concentrations are maintained over time, because pesticides inhibit the enzymes involved in reducing its levels. Thus, the alteration of Ca^2+^ levels can lead to the activation of various signaling pathways that generate oxidative stress, neuroinflammation and, finally, neuronal death. In this Special Issue, Costas-Ferreira and Faro summarize the main mechanisms of pesticides’ actions on neuronal Ca^2+^ homeostasis (Figure 2) [35].

VGCCs are divided into high-voltage-activated L-type (Cav1.1, Cav1.2, Cav1.3, and Cav1.4), P/Q-type (Cav2.1), N-type (Cav2.2), and R-type (Cav2.3) channels because they are activated by relatively large depolarizations, as well as low-voltage-activated T-type (Cav3.1, Cav3.2, Cav3.3) channels, because they are activated by relatively small depolarizations. Among VGCCs, Cav1.2 is the dominant type in the cardiac working muscle, while Cav1.3 and Cav3.2 are dominant in pacemaker (nodal) cells. Ca^2+^ influx through Cav1.2 channels is regulated by the negative feedback mechanism, known as Ca^2+^-dependent inactivation (CDI), as well as the positive feedback mechanism, known as Ca^2+^-dependent facilitation (CDF) [36]. Calmodulin (CaM) is thought to play an important role in both CDI and CDF. Furthermore, CaM may have roles in channel trafficking and clustering/multimerization. In this Special Issue, Kameyama et al. reviewed the details of cardiac Cav1.2 channels’ regulation by CaM [37]. The direct interactions with CaM, a Ca^2+^-binding protein, causes Ca^2+^-dependent facilitation (CDF) and the inactivation (CDI) of the Cav1.2 channels. Ca^2+^-free CaM (apoCaM) also contributes to the regulation of Cav1.2 channels. A role of apoCaM in the channel ‘rundown’ phenomena and the related repriming of channels, and CDF, as well as the role of Ca^2+^/CaM in CDI, has been identified. Furthermore, CaM indirectly affects channel activity by activating CaM-dependent enzymes, such as CaM-dependent protein kinase II and calcineurin (a CaM-dependent protein phosphatase). In addition, Cav1.2 channels’ trafficking are regulated through the intrinsic properties of isoforms of the α1 subunit of the channel, the associated auxiliary subunits, and regulatory proteins such as CaM. However, Kameyama concluded that the role of CaM in the trafficking of Cav1.2 channels remains controversial and further studies are required.

The heart possesses huge amounts of mitochondria which serve to supply ATP via oxidative phosphorylation, meeting the cardiac energy demand. The mitochondrial Ca^2+^—one of the key factors of mitochondrial energetics—is strictly maintained within an appropriate range [38]. The mitochondrial Ca^2+^ in cardiomyocytes is balanced by an influx via the Ca^2+^ uniport activity and by an efflux via the Na^+^-Ca^2+^ exchange or the H^+^-Ca^2+^ exchange activities in which the former plays the major part [39,40]. The mitochondrial Na^+^-Ca^2+^ exchanger, NCLX, was reported to supply Ca^2+^ to the sarcoplasmic reticulum (SR)/ER, thereby modulating various cellular functions such as the rhythmicity of cardiomyocytes and cellular Ca^2+^ signaling upon antigen receptor stimulation and chemotaxis in B lymphocytes; however, there is little information on the spatial relationships of NCLX with SR Ca^2+^ handling proteins, and their physiological impact. In this Special Issue, Takeuchi and Matsuoka examined the tissue, focusing on the interaction of NCLX with an SR Ca^2+^ pump (SERCA) in cardiomyocytes [41]. A bimolecular fluorescence complementation assay using HEK293 cells revealed that the exogenously expressed NCLX was localized in close proximity to four exogenously expressed SERCA isoforms. Immunofluorescence analyses of isolated ventricular myocytes showed that the NCLX was localized to the edges of the mitochondria, forming a striped pattern. The co-localization coefficients in the super-resolution images were higher for NCLX–SERCA2 than for the NCLX–RyRs and NCLX–Na^+^/K^+^ ATPase α-1 subunit, confirming the close localization of endogenous NCLX and SERCA2 in cardiomyocytes. The mathematical model implemented with the spatial and functional coupling of NCLX and SERCA reproduced the NCLX inhibition-mediated modulations of SR Ca^2+^ reuptake in HL-1 cardiomyocytes well. These results indicated that NCLX and SERCA are spatially and functionally coupled in cardiomyocytes. The functional coupling plays pivotal roles in the SR Ca^2+^ dynamics and in the generation of automaticity in cardiomyocytes.

The dysregulation of store-operated Ca^2+^ entry (SOCE) promotes cancer progression by changing Ca^2+^ levels in the cytosol or the ER. Stromal interaction molecule 1 (STIM1), which initiates SOCE by sensing severe ER Ca^2+^ store depletion, is upregulated in several types of cancer and responsible for cancer cell migration, invasion, and metastasis. In this Special Issue, Lin et al. [42] investigated the impact of STIM1-mediated SOCE on the turnover of focal adhesion (FA) and cancer cell migration. They overexpressed the wild-type and constitutively active or dominant negative variants of STIM1 in an osteosarcoma cell line, expecting that STIM1-mediated Ca^2+^ elevation may increase cell migration. However, constitutively active STIM1 dramatically increased the Ca^2+^ influx, calpain activity, and turnover of FA proteins, such as the focal adhesion kinase (FAK), paxillin, and vinculin, which impede the cell migration ability. In contrast, dominant negative STIM1 decreased the turnover of FA proteins as its wild-type variant compared to the cells without STIM1 overexpression while promoting cell migration. These results suggest that cancer cells need an appropriate amount of Ca^2+^ to control the assembly and disassembly of focal adhesions by regulating calpain activity. On the other hand, overloaded Ca^2+^ results in excessive calpain activity, which is not beneficial for cancer metastasis.

Papers in this Special Issue entitled “Calcium Channels and Calcium-Binding Proteins” demonstrated broad effects of Ca^2+^ dynamics controlled by various mechanisms for cell and organ-specific action.

## Figures and Tables

**Figure 1:**
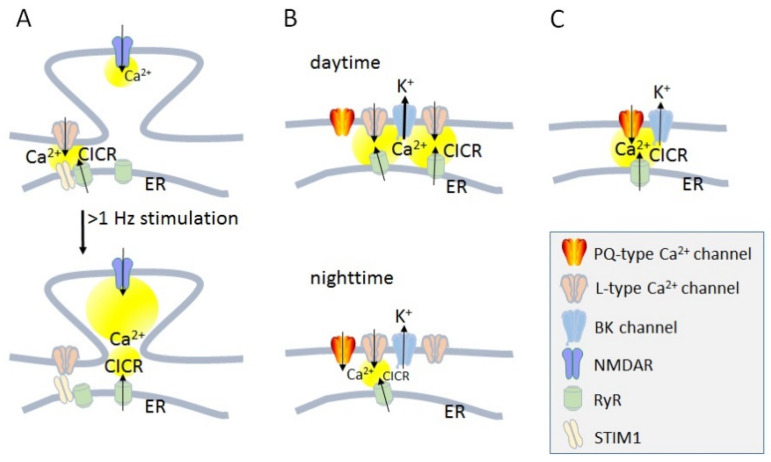
Calcium dynamics with the activation of Ca^2+^ channels and Ca^2+^-induced Ca^2+^ release in neurons. (**A**) Ca^2+^-induced Ca^2+^ release (CICR) and activity-dependent regulation of postsynaptic voltage-gated Ca^2+^ channels (VGCC). (**B**) Circadian regulation of dendritic Ca^2+^ dynamics. (**C**) Dual Ca^2+^ nanodomain in Soma. Yellow circles indicate predicted Ca^2+^ elevation. Reproduced with permission from Ref. [1]; published by Elsevier, 2020.

**Figure 2:**
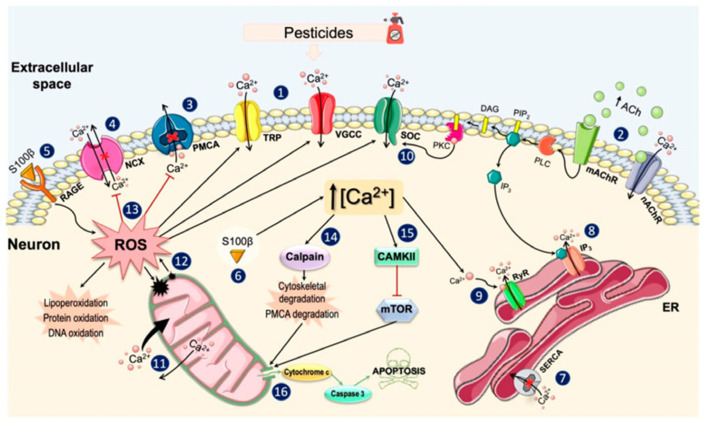
Main mechanisms of action of pesticides on neuronal Ca^2+^ homeostasis. Reproduced from Ref. [35]. Published in this Special Issue by MDPI, 2021. Exposure to pesticides induces a series of changes in the plasma membrane that include: (**1**) the opening of the VGCCs (especially the L- and T-types) and some TRP channels, which allows the Ca^2+^ influx and enhances the membrane depolarization; (**2**) the activation of nicotinic acetylcholine receptors (nAChR) and muscarinic acetylcholine receptor (mAChR) through increasing the availability of acetylcholine and/or by binding directly to these receptors; (**3**) the inhibition of plasma membrane Ca^2+^-ATPase, the main Ca^2+^ extrusion mechanism; (**4**) alterations in the Na^+^/Ca^2+^ exchanger, completely inhibiting its activity or activating its reverse mode. Pesticides also increase the S100*β* levels, which (**5**) bind to the receptor for advanced glycation end products in the extracellular side and favor the production of reactive oxygen species (ROS) and (**6**) increase the Ca^2+^ levels in the intracellular medium. In the cytosol, pesticides induce the depletion of the endoplasmic reticulum (ER) Ca^2+^ reserves by (**7**) inhibiting the sarcoplasmic (endoplasmic) reticulum Ca^2+^-ATPase, responsible for sequestering Ca^2+^; (**8**) stimulating the inositol 1,4,5-trisphosphate (IP_3_)-induced Ca^2+^ release; and (**9**) via Ca^2+^ release through RyRs stimulated by cytosolic Ca^2+^. When the Ca^2+^ content of the ER begins to decline, the protein kinase C (PKC) stimulates the influx of Ca^2+^ through the store-operated channels (SOC) (**10**) and the mitochondria assume the role of the Ca^2+^ reservoir, rapidly accumulating large amounts of Ca^2+^ and slowly releasing it (**11**). The overload of Ca^2+^ in the mitochondria increases ROS levels and its release to the cytosol (**12**), where they enhance the [Ca^2+^]_i_ by stimulating Ca^2+^ channels and inhibiting their expulsion mechanisms, damaging lipids, cell proteins, and DNA (**13**). Increases in ROS and Ca^2+^ levels activate calpains and Ca^2+^/calmodulin-dependent protein kinase II (CAMKII). Calpains induce the degradation of elements of the cellular cytoarchitecture (**14**), while CAMKII inhibits mammalian or mechanistic targets of rapamycin (mTOR) (**15**). These two pathways can ultimately cause the release of pro-apoptotic factors from the mitochondria, finally leading to cell death (**16**).

## Abbreviations

CDF	Ca^2+^-dependent facilitation
CDI	Ca^2+^-dependent inactivation
CICR	Ca^2+^ induced Ca^2+^ release
ER	Endoplasmic reticulum
FA	Focal adhesion
KCa	Ca^2+^-activated potassium
LOT	Lateral olfactory tract
LTCCs	L-type calcium channels
LTP	Long-term potentiation
LTD	Long-term depression
NCLX	Mitochondrial Na^+^-Ca^2+^ exchanger
NCX	Na^+^/Ca^2+^ exchanger
NKA	Na^+^/K^+^-ATPase
NMDARs	N-methyl-D-aspartate receptors
PC	Piriform cortex
PMCA	Plasma membrane Ca^2+^-ATPase
RIM	Rab3-interacting molecules
ROS	Reactive oxygen species
RyRs	Ryanodine receptors
SCN	Suprachiasmatic nucleus
SERCA	SR Ca^2+^ pump
SOCCs	Store-operated Ca^2+^ channels
SR	Sarcoplasmic reticulum
STIMs	Stromal interaction molecules
VGCC	Voltage-gated Ca^2+^ channels
